# Improving glycemic control in critically ill patients: personalized care to mimic the endocrine pancreas

**DOI:** 10.1186/s13054-018-2110-1

**Published:** 2018-08-02

**Authors:** J. Geoffrey Chase, Thomas Desaive, Julien Bohe, Miriam Cnop, Christophe De Block, Jan Gunst, Roman Hovorka, Pierre Kalfon, James Krinsley, Eric Renard, Jean-Charles Preiser

**Affiliations:** 1 Department of Mechanical Engineering, Centre for Bio-Engineering, University of Canterbury, Christchurch, New Zealand; 2 GIGA In-Silico Medicine, University of Liège, Liège, Belgium; 3 Medical Intensive Care Unit, Lyon-Sud University Hospital, Pierre-Bénite, France; 4 ULB Center for Diabetes Research, and Division of Endocrinology, Erasme Hospital, Université Libre de Bruxelles, Brussels, Belgium; 5 Department of Endocrinology, Diabetology and Metabolism, Antwerp University Hospital, Edegem, Belgium; 6 Clinical Division and Laboratory of Intensive Care Medicine, Department of Cellular and Molecular Medicine, KU Leuven, Leuven, Belgium; 7 University of Cambridge Metabolic Research Laboratories, Level 4, Wellcome Trust–MRC Institute of Metabolic Science, Addenbrooke’s Hospital, Cambridge, UK; 8 Service de Réanimation polyvalente, Hôpital Louis Pasteur, CH de Chartres, Chartres, France; 9 Division of Critical Care, Department of Medicine, Stamford Hospital, Columbia University College of Physicians and Surgeons, Stamford, CT USA; 10 Department of Endocrinology, Diabetes, Nutrition, and Institute of Functional Genomics, CNRS, INSERM, Montpellier University Hospital, University of Montpellier, Montpellier, France; 11 Department of Intensive Care, Erasme Hospital, Université Libre de Bruxelles, route de Lennik 808, 1070 Brussels, Belgium

**Keywords:** Glycemic control, Endocrine function, Artificial pancreas, Modeling, Model based, Validation, Virtual patient, In silico, Review

## Abstract

There is considerable physiological and clinical evidence of harm and increased risk of death associated with dysglycemia in critical care. However, glycemic control (GC) currently leads to increased hypoglycemia, independently associated with a greater risk of death. Indeed, recent evidence suggests GC is difficult to safely and effectively achieve for all patients. In this review, leading experts in the field discuss this evidence and relevant data in diabetology, including the artificial pancreas, and suggest how safe, effective GC can be achieved in critically ill patients in ways seeking to mimic normal islet cell function. The review is structured around the specific clinical hurdles of: understanding the patient’s metabolic state; designing GC to fit clinical practice, safety, efficacy, and workload; and the need for standardized metrics. These aspects are addressed by reviewing relevant recent advances in science and technology. Finally, we provide a set of concise recommendations to advance the safety, quality, consistency, and clinical uptake of GC in critical care. This review thus presents a roadmap toward better, more personalized metabolic care and improved patient outcomes.

## Background

Hyperglycemia is prevalent in critical care, caused by a complex interaction of multiple feedback loops associated with inflammation as a result of immune responses, counter-regulatory responses, and high blood glucose itself [1, 2]. Hyperglycemia is exacerbated by unsuppressed endogenous glucose production [1], some medications (steroids/catecholamines), and high exogenously administered nutrition [3]. There is also suppression/loss of pancreatic insulin secretion, and loss of sensitivity to insulin, resulting in reduced insulin-mediated glucose uptake. Thus, the question arises of whether there is a need for an “artificial pancreas” or another form of closed-loop, highly personalized glycemic control (GC) in critical care, similar to those emerging in type 1 diabetes [4].

To date, blood glucose control to obtain metabolic homeostasis has given mixed results in clinical trials in critically ill patients. Initial results of reduced morbidity and mortality with tight GC [5–9] could not be reproduced in large prospective trials [10–12]. More recent randomized trials using more advanced protocols have not altered the general direction [13]. However, recent observational analysis [14] indirectly supports the concept that altered glycemia, and not the underlying patient or metabolic condition, causes the increase in mortality, and thus GC is important and needs to be performed well. Thus, using higher blood glucose targets to ensure that hypoglycemia is avoided may not be good enough, reopening some of the debate on GC in terms of how to provide consistent, safe, effective management. However, significant issues prevent safe, effective GC, clinically, scientifically, and technologically—this short state-of-the-art review addresses these issues, resulting in a set of recommendations.

An effective GC protocol or artificial pancreas should provide insulin similar to a normal subject. In normoglycemic individuals, a hyperbolic relationship exists between insulin sensitivity and insulin secretion, leading to the disposition index concept, a measure of pancreatic beta cell function adjusted for insulin sensitivity [15]. Studies show that pancreatic function is deranged in critically ill patients [1] displaying similarities to type 2 diabetes [16, 17], namely insufficient insulin secretion in a context of decreased insulin sensitivity. The disposition index is therefore reduced, as in patients with diabetes, as a result of inflammatory and stress hormones. In both critical illness and type 2 diabetes, hyperglycemia results from reduction in the first-phase insulin response [18, 19]. However, the associations between gene markers and outcome identified in type 2 diabetes have not been found in critical illness [20], and pancreatic function changes in critical illness are not associated with obesity or diet [21]. Thus, type 2 diabetes and critical care hyperglycemia can both feature reduced insulin sensitivity, reduced insulin clearance, and insufficiently increased insulin secretion, with resulting hyperglycemia. These effects can appear more severe over a very short term in critically ill patients and have different causes. In both conditions, the pancreas cannot provide the necessary insulin nor fully suppress hepatic glucose production.

The pancreas is linked to a continuous, accurate glucose “sensor” to guide insulin secretion and control. However, an equivalent “exogenous” sensor is lacking, despite emerging continuous glucose monitors [22]. Essential requirements for an artificial pancreas in the ICU include: accurate real-time or high-frequency continuous glucose monitoring [23]; continuous intravenous insulin infusion; and an adequate algorithm that automatically drives the intravenous insulin pump. A closed-loop system with accurate continuous glucose monitoring and computer-assisted titration of insulin based on glucose measurements could permit tight GC without increasing hypoglycemia and nursing staff workload.

However, there are significant hurdles to creating an effective artificial pancreas, both scientifically and technologically. A 2017 ISICEM Working Group addressing the artificial pancreas in critical care delineated and reviewed the recent research addressing these hurdles. The subsequent sections define the clinical hurdles in this field, which we use to define key scientific and technical needs. We then provide a short state-of-the-art overview for each need, leading to a set of recommendations for improving GC in the intensive care unit (ICU).

## Clinical hurdles

Mixed results from GC clinical studies left the field questioning the weight of data correlating high glucose, high glycemic variability, and increased hypoglycemia from GC with increased morbidity and mortality. The result has led to a recommendation for a range of “soft” targets, essentially permitting hyperglycemia of 8.0–10.0 mmol/l to avoid hypoglycemia [24], despite evidence associating increased time in tight/intermediate glycemic bands with improved outcomes [25–27]. Hence, the field is at a crossroads between permissive hyperglycemia and the inability to safely, effectively control glycemia [14].

Indeed, safe strict GC has proved elusive [5, 7, 9]. Diverging results of randomized controlled trials on GC may be explained by important methodological differences between trials, compliance, experience, motivation, and/or protocol. Studies showing a positive impact of tight GC used accurate glucose monitors and were in general more successful in achieving the blood glucose target (e.g., [28]). In subsequent nonconfirmatory studies, the time in the target range was smaller. Achieving > 50% time in blood glucose bands of 4.0–7.0 and/or 5.0–8.0 mmol/L for 90% or more of patients is considered an index of good performance [25–27]. Although only some patients may benefit from good control [6], an inability to identify them directly requires safe, tight control to be delivered to all patients.

Recent work suggests that glycemic outcomes and their association with morbidity and mortality outcomes are a function of the quality of control applied [14]. While this analysis needs repeating over larger cohorts for certainty, it indicates that current acceptance of permissive hyperglycemia may carry risk and that GC has a role to play if it can be delivered with greater safety and performance. In particular, without better safety from hypoglycemia it is difficult to assess whether the effects of strict GC are beneficial. Personalized, patient-specific GC, potentially including recognition of diabetic status or other factors, thus offers a route to GC that is safe and effective for all patients [29], which in turn would enable new trials to better assess benefit.

Personalized or patient-specific GC transforms bedside GC data into accurate representations of the patient-specific metabolic state. Patient-specific models can be used to safely design GC algorithms in silico, to minimize risk and avoid the mixed results arising from trial-and-error clinical protocol design.

Further clinical hurdles include ergonomics and workload [30, 31]. Poor ergonomics lead to noncompliance, and thus poor, inconsistent control and outcomes [30]. Workload, and thus the potential need for automation, further hinders clinical uptake. Emerging technologies with full automation of sensors and/or pumps offer first opportunities to examine the potential of automation on workload and GC quality [32].

A final hurdle is the inability to fully learn from prior efforts. Many studies do not report results in the same way, with numerous different metrics [33]. Those metrics reported often do not allow reconstruction of the full results distribution, limiting complete understanding. Hence, it is difficult to extract significant, generalizable lessons from many studies.

## Needs statement and goals

These clinical hurdles yield four main needs:

A. To accurately understand patient-specific, real-time metabolic status.
B. To develop a validated means to design GC methods to fit clinical practice.
C. To create the right type of safe, effective GC, providing good control for all patients with an acceptable workload.
D. To determine the correct set of metrics to evaluate GC safety and performance, which is generalizable across studies.

These needs lead to three main goals to create a step-change in GC capability. Specifically, these are as follows:

1. Model and (virtual patient) simulator: addressing needs A and B using in-silico methods to safely design protocols and, in use, to personalize care to patient-specific metabolic status.
2. Control (approach): addressing need C, where many approaches exist, both model-based and clinically derived, but few have provided consistent safety and performance.
3. Metrics: directly addressing need D and the difficulty in comparing the safety and performance across studies and protocols to derive the lessons learned and advance understanding.

**Fig. 1:**
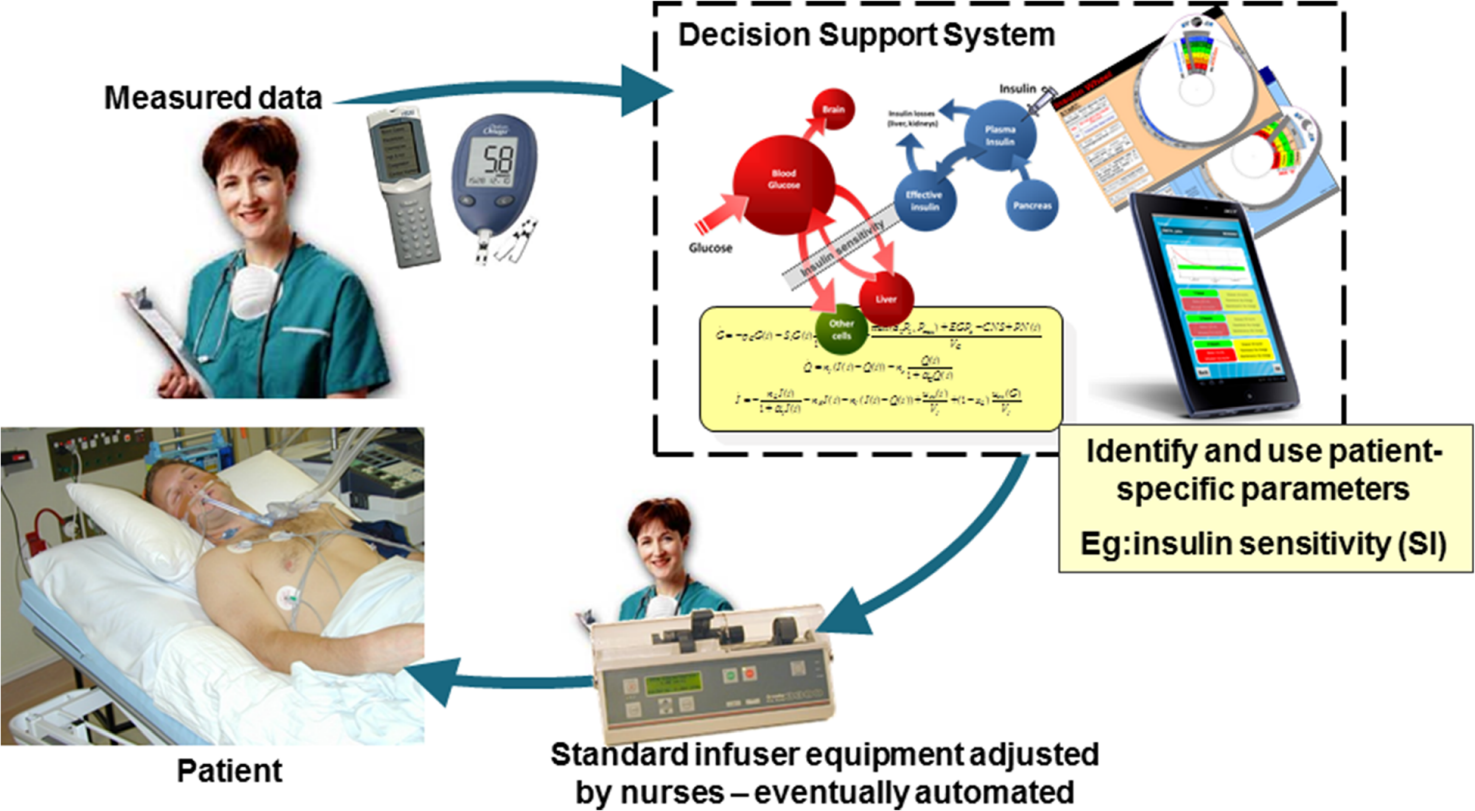
Model-based decision support to mimic the human pancreas with a nurse in the loop, but eventually automated. Measurements and other data are given to a decision support system that identifies patient-specific information, such as insulin sensitivity, to personalize the model. A control protocol uses these data to generate personalized recommendations for patient care. Change the model-based decision support with a clinical protocol and you would have standard care

**Fig. 2:**
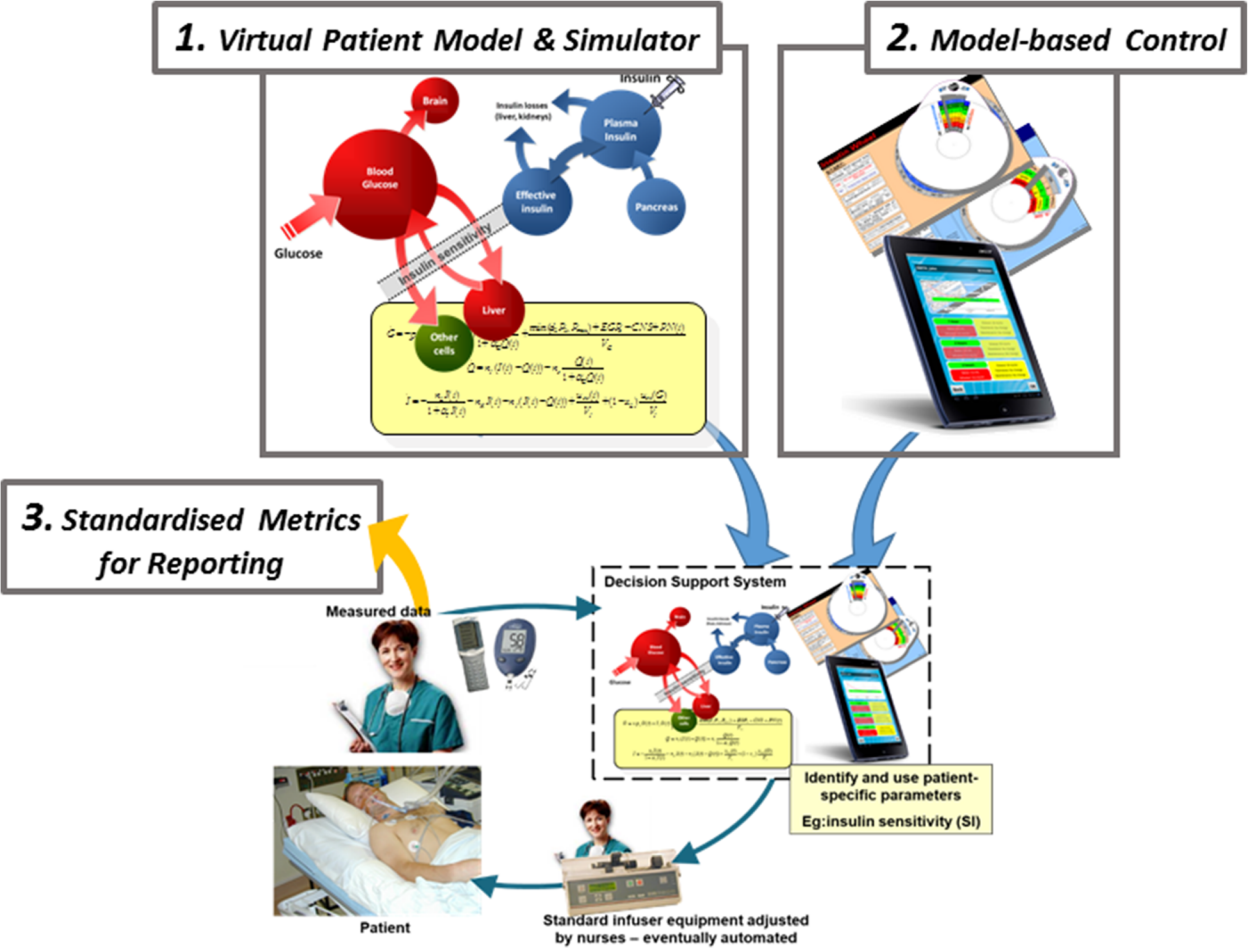
Three main needs identified related to the overall model-based control loop of Fig. 1

Figure 1 outlines the current nurse in the loop approach to model-based GC. Replacing a model-based decision support method with a standard clinical protocol or nurse-driven care shows the loop used in today’s standard care. Figure 2 shows how the three identified areas of need fit into this feedback loop in Fig. 1, and thus outline the overall review, as well as showing how these elements fit into clinical care.

### Models and virtual patient simulators

#### Metabolic system models

There are several models of human metabolism, predominantly deterministic compartment models with specific terms representing the relevant physiological behaviors [34], as well as black-box or data-driven models with no direct physiological analogy or relevance. Given the need to identify the patient-specific metabolic status with limited data for personalized GC, this section focuses on less complex models, where the minimum fundamental model inputs to simulate and/or personalize these models are the insulin and nutrition given, and blood glucose measurements, but can be more extensive. However, no discussion of possible models is complete due to the large number of models that have been studied, so this section focuses on those used in critical care and for creating virtual patients, specifically:

- ICU Minimal Model [35].
- Glucosafe model [36].
- Cambridge model [37].
- ICING model [38, 39].
- UVA/Padova model [40].

Each model has been used to create in-silico virtual patients, and has thus moved beyond limited clinical experiments toward engineering design tools to optimize care.

The ICU Minimal Model is based on the Bergman Minimal Model [41], and was used to design the LOGIC controller [42]. It is the least physiologically relevant, with a minimal number of terms aggregating all glucose appearance and disappearance routes, and thus the least specific in terms of how the model terms directly relate to specific physiological phenomena. Identifying an insulin sensitivity parameter derived from two model parameters makes it patient specific. It has been used clinically and has shown improved control over nurse-managed GC, a final form of validation [43].

Glucosafe is derived from an earlier form of the ICING model and a diabetes model [44]. Insulin sensitivity is identified from data and used to create virtual patients [36]. Glucosafe is more physiologically relevant than the ICU Minimal Model. However, it has demonstrated limited performance in pilot GC trials [45].

The Cambridge model is highly physiologically relevant [37], was developed from frequently sampled bedside data collected in the ICU, and has been used to simulate and design GC in critical care. It uses identified time-variant insulin sensitivity to guide care and mitigate errors [46].

The ICING model also has high physiological relevance, has an insulin sensitivity parameter identifiable from bedside data, and has been used in virtual patients [39, 47], GC design [48, 49], and real-time GC [49, 50].

The UVA/Padova simulator was developed for type 1 diabetes [40]. This is not a single model, but a collection of submodels with very high physiological relevance and detail. However, a given insulin sensitivity or other key parameters cannot necessarily be identified from bedside data, limiting it to simulations of generic patients, and it is thus not validated for clinical use.

While this brief overview is not exhaustive, it clearly highlights the need for “valid models” meeting three main criteria. First, an identifiable insulin sensitivity parameter capturing patient-specific glucose responses to insulin/nutrition inputs to enable personalization. Second, a large enough degree of physiological relevance to ensure that the identified parameter(s) accurately capture patient-specific behavior so they can be used to design and/or guide care. Third, rigorous validation via use in the design and/or implementation of safe, effective GC. These issues are inter-related, and thus reviewed in subsequent sections in terms of the use of these models as virtual patients to design and guide care, and their subsequent use in guiding GC.

#### Virtual patients and simulators

Given a metabolic model and the required insulin, nutrition, and blood glucose data to identify key parameters to personalize the model, a virtual patient might be created. A virtual patient is built from the combination of a metabolic model and clinical patient data, creating an in-silico representation of that patient on which new treatment approaches might be tested, either to create a new protocol and/or in real time at the bedside to guide care safely and effectively. Thus, a metabolic model captures behavior, and a virtual patient simulator uses that model with clinical data to mimic patient behavior to design new therapeutic approaches.

Virtual patients come in two types:

- Generic/cohort specific: created from clinical data or model parameter choices, they capture overall patient types or behaviors.
- Clones/patient specific: created directly from clinical data, they capture the actual patient-specific response of a specific patient, and have thus created an in-silico model “clone” of the patient from their clinical data, which responds and acts in the same way as the actual patient.

Generic virtual patients use a selection of clinical data to fit general, cohort-wide model parameters, which are perturbed to create realistic cohorts. Simulations are only valid for predicting cohort-level responses, such as average glycemia and variability, safety from hypoglycemia, and required insulin/nutrition inputs. However, specific virtual patient results may not be realistic. Examples include the Cambridge simulator [37, 51], the Zealand simulator [52], the UVA/Padova simulator [53], the Medtronic simulator [54], and the ICU Minimal Model simulator [35] in diabetes. The Cambridge model has captured the central tendency of an ICU cohort [55].

Cloning specific patients from their data to create an in-silico, computer model mimic is rarer. The UVA model was used for single-day clones [56] and the Cambridge model in multiday clones [37, 51, 55] in critical care. Glucosafe [36] was used to create multiday ICU patient clones for clinical GC [45]; however, the clinical outcomes did not match reality as well as desired. Finally, the ICING model and predecessors were used to create multiday virtual patient clones to design and implement multiple GC protocols. Predicted cohort-level glycemic results were matched by subsequent clinical results (e.g., [49]) with good correlation of median per-patient glucose levels, indicating that the cloned virtual patients were a good representation [39, 47].

The primary concern in creating virtual patients to design GC protocols is whether results of the virtual patient simulations accurately predict clinical safety, performance, and workload when a given protocol is implemented clinically. Accurate prediction of what happens when a model is implemented clinically provides assurance that the model and virtual patient simulator have the fidelity to design a protocol with confidence in the resulting safety, performance, and workload, as well as the fidelity to be used in real-time GC in decision support. Only the Cambridge and ICING simulators have delivered good results in this regard. To date, only the ICING and Cambridge models have been validated using independent cohorts and protocols [39, 47, 55].

A GC protocol design with virtual patients can be highly effective at predicting GC safety and performance in clinical use. It can thus limit poor results, particularly from easily avoidable protocol design errors. The main hurdle is the low number of validated models available.

### Control

#### Glycemic control and decision support

GC using semi-automated (human-in-the-loop) or fully automated insulin/nutrition dosing is a long-term research area. Increasing automation brings technological risk, but significantly reduces workload, human error, and compliance risks. However, both approaches use the same GC protocol, so this section focuses on the two main means of applying control. Specifically, looking backward using a feedback approach, and looking forward using a predictive approach. Both approaches can be personalized using models, although personalization is typically only used in predictive methods.

#### Feedback control

Feedback control takes blood glucose measurements and other inputs, and suggests interventions in response to the current state and/or changes since the last measurement/intervention.

The most well-known feedback control systems are paper-based or computerized clinical protocols, typically ranging from simple static sliding scales to more complex dynamic scales. Static scales adjust insulin based on the blood glucose level alone. They are the simplest systems and offer no adaptability, with a “one size fits all” approach, thus essentially assuming a linear effect of increasing insulin with all patients assumed to have similar insulin sensitivity [57]. Dynamic scales change the insulin dose, based on the blood glucose range the patient is in and prior responses. The Leuven [7], Krinsley [9], and NICE-SUGAR [11] protocols are dynamic scales. However, many do not directly consider or control for nutrition, creating significant variation in responses [58].

Proportional-derivative (PD) and/or proportional-integral-derivative (PID) controllers operate like complex dynamic scales. Each insulin intervention is based on the error in blood glucose from the target (P), the rate of change of that error (D), and the area under the error curve (I). The P, I, and D gains multiply these values using the most recent blood glucose measurements to calculate the insulin dose. PID control and similar dynamic scales in critical care include the Endotool (Monarch Medical, USA) [59], GRIP [60], Glucommander (Glytec Systems, USA) [61], GlucoCare (Pronia Medical, USA) [62], and GlucoStabiliser (Medical Decision Network, USA) [63] systems commercially and several research tools [13, 14, 64–66]. Advantages include simplicity and easy automation. Disadvantages include lack of significant patient specificity, which is only seen indirectly via patient-specific glucose response to insulin, and lack of input about nutrition or (often) prior insulin doses. These disadvantages can lead to greater variability across patients in larger, heterogeneous cohorts.

Overall, the performance of clinical protocols or dynamic scales is better than that of static sliding scales, but is exceeded by PID control. More successful protocols include modifications around insulin on board, patient-specific response, and/or physiological inputs. While these models improve safety and performance, they have similar increased clinical workload to systems without automation.

Unlike all other feedback controllers, SPRINT [5] controls nutrition. Developed using virtual trials [47, 48], this protocol modulates insulin and nutrition using implicitly calculated patient-specific insulin sensitivity. SPRINT is thus more patient specific and was the only protocol associated with reduced glycemia, mortality, and hypoglycemia. The main message from this study [5] was the need to consider nutrition in safe, effective GC, which model-based, predictive controllers can do.

#### Predictive control

Predictive control requires a model that takes current measurements and other inputs, identifies patient-specific model parameters to personalize the control, and uses the patient-specific model to predict the outcome of insulin/nutrition interventions to optimize glycemic performance. The model is used directly in the control loop.

Although several model-based and predictive controllers have been tested in short or limited trials, only a few have been used regularly in major trials [42] or as a standard of care [50, 67]. There are two essential approaches: target to value (TTV), GC to a specified glycemic target value; and target to range or risk (TTR), GC to a specified risk of hypoglycemia or hyperglycemia.

The LOGIC-Insulin system is a TTV approach and nutrition is clinically set and not controlled. The randomized, single-center LOGIC-I trial [42] compared standard care at a unit with a good reputation for nursing-led GC to LOGIC-Insulin model-based care with very good results. This performance was confirmed in a multicenter trial [43], but it is not yet a standard of care in the original study unit.

The TTV eMPC (B. Braun, Germany) [46] has been used in several trials [66–69], controlling insulin infusions and leaving nutrition clinically set. Compared to standard care, eMPC does well [66, 69]. Comparisons across centers and cohorts show similar, but not identical, performance [67, 68]. Workload is 14–18 measurements/day, and thus higher than standard care. Thus, eMPC provides improved care and safety, but increases potential workload. It is used regularly in some ICUs.

STAR is the only TTR system and controls both insulin and nutrition input [49, 70], using risk-based stochastic forecasting [71]. It is the only GC system to directly account for future variability. STAR has good performance and safety, including high times in intermediate glycemic bands, approaching 80% with 10–13 measurements/day [50]. It generalized well, with almost identical glycemic outcomes across very different cohorts and ICUs [50]. Notably, only eMPC and LOGIC-Insulin have shown similar generalizability.

All three model-based systems noted have very low rates of hypoglycemia (< 5% by patient) and are very generalizable. These results indicate that predictive, model-based methods can overcome many of the hurdles that have hindered several other trials.

In summary, at this time, in addition to PID feedback systems used in some hospitals in the USA [61–63], three model-based predictive GC methods have proved reliable over multiple patient types and centers. Two methods are used as standards of care in multiple hospitals, and one of these has a neonatal ICU version [72] used as standard care and in a randomized trial [73]. Both methods consider nutrition, although only one controls it, which may account for some differences in performance. Overall, successful model-based GC takes into account patient-specific factors. The patient’s metabolic state evolves over the course of their illness, and thus this approach provides adjustments as needed within a given patient, as much as across patients.

### Metrics and a recommended minimum standard

Glycemic reporting metrics are diverse [33]. Given significant analyses linking organ failure and mortality to time in target bands [6, 25, 27], time-in-band percentages should be standard cohort and patient-level metrics. Unlike the median (IQR) or mean (standard deviation), time in range to a given threshold (e.g., patients with > 50% blood glucose within 4.0–7.0 mmol/L) captures the central tendency (mean or median) and variability (IQR, standard deviation). Higher thresholds indicate tighter control for a given cohort or patient in a given blood glucose range.

To assess any range of the several possible, the cohort cumulative distribution function (CDF) or s-curve should be reported. This approach enables any time in range to be assessed. These CDFs can be aggregated for each patient, and provide a data set with every possible time-in-range outcome shown, from which comparisons can be readily made.

For safety, the percentage blood glucose < 3.9 mmol/L and the percentage of patients who have one or more episodes of blood glucose < 2.2 mmol/L should be reported.

Workload can be minimally reported as measurements/day, because measurements are a primary source of workload [31], but could add other GC-related effort [30].

All of these metrics would include all patients for all days of stay, from the start of GC. These recommendations provide a minimal data set for regular and standardized comparison across cohorts, trials, and publications. Any other analyses or data reporting would be additional to this minimal set.

## Summary recommendations

Based on this overview and analysis of the current state-of-the-art for GC in critically ill patients, the following recommendations are made to advance the safety, quality, consistency, and clinical uptake:

1. Patient-specific model-based GC including closed-loop systems, increasingly enabled by the penetration of computational technology into the ICU, can improve the quality of GC:
  - models should be self-validated and cross-validated;
  - initial assessment and optimization in validated virtual trials should be considered for new GC methods;
  - final validation of safety and performance must come in clinical (pilot and/or randomized) trials.
2. GC could consider nutrition as an input certainly, and possibly as a controlled clinical input set against international guideline goal feeds. No GC care should be “carbohydrate blind”, even though closed-loop systems may operate without nutritional input.
3. No GC trial should be performed without first conducting a pilot trial in each involved ICU, which demonstrates that safe, effective GC can be obtained to the desired level for 80% or more of patients, unless an algorithm is used that has already been validated in a multicenter context. Each potential trial center must prove it can safely and consistently achieve the desired level of GC stated in the trial plan.
4. To enable comparison and analysis, all GC reporting should have a minimum standardized set of data reporting performance, safety, and workload, including:
  - performance—time in desired target band;
  - safety—number of patients experiencing severe hypoglycemia (blood glucose < 2.2 mmol/L) or percentage blood glucose < 3.9 mmol/L;
  - workload—average number of staff-taken or automated (if applicable) glucose measurements per patient per day (standard deviation) or median (IQR);
  - performance (optional)—CDFs of cohort glycemia, which provide all possible time-in-target ranges, enabling far easier comparison;
  - performance (optional)—CDFs of per-patient glycemia.
5. ICU clinicians should press for increasing automation and access to sensor and infusion pump data for independent processing of GC methods to increase safety and reduce workload.

## Conclusions

Glycemic control has proven difficult to safely and effectively achieve for all patients, where modeling and model-based methods have offered a potentially significant avenue to achieving safe, effective control. In this review, leading experts discuss this evidence, report relevant reports from medicine and engineering, and suggest how safe, effective GC can be achieved in critically ill patients, ultimately seeking to mimic pancreatic function. The review concludes with concise recommendations to advance the safety, quality, consistency, and clinical uptake of GC in critical care, providing a roadmap toward better, more personalized metabolic care and improved patient outcomes.

## Abbreviations

CDF: Cumulative distribution function
GC: Glucose control
ICU: Intensive care unit
TTR: Target to range or risk
TTV: Target to value
